# Magnitude and associated factors of common mental disorders among pregnant women during COVID-19 in Ethiopia: A systematic review and meta-analysis

**DOI:** 10.1097/MD.0000000000041842

**Published:** 2025-03-14

**Authors:** Aragaw Asfaw Hasen, Ahmed Adem Mohammed, Mekash Ayalew Mohammed, Abubeker Alebachew Seid

**Affiliations:** a Department of Statistics, College of Natural and Computational Sciences, Samara University, Semera, Ethiopia; b Department of Nursing, College of Medicine and Health Sciences, Samara University, Semera, Ethiopia; c Department of Mathematics, College of Natural and Computational Sciences, Samara University, Semera, Ethiopia.

**Keywords:** anxiety, COVID-19, depression, Ethiopia, mental health, pregnancy, stress, systematic review and meta-analysis

## Abstract

**Background:**

Pregnant women have multiple psychological distresses and are psychologically fragile. In Ethiopia due to COVID-19 anxiety, depression and stress among pregnant women were increased. This study aimed to provide comprehensive data on the prevalence and associated factors of common mental disorders during COVID-19 in Ethiopia.

**Methods:**

Data were searched from PubMed, Google Scholar, and African Journals Online from the December 2019 to August 2024. Two researchers extracted the data and accomplished the methodological quality valuation independently. Random-effect model used to estimate the pooled effect size and *I*^2^ and *Q*-statistic were used to check heterogeneity. Stata 14.0 (Stata Corp, College Station, Texas, USA) was used for statistical analysis.

**Results:**

Eleven studies were included. From 6 studies the pooled prevalence of anxiety was 47% (95% confidence interval [CI] = 0.37 to 0.57, *I*^2^ = 96.00%, *P* < .001). Five studies reported about depression and the pooled prevalence was 32% (95% CI = 0.22 to 0.42, *I*^2^ = 96.00%, *P* < .001). Four studies reported about stress and the pooled prevalence was 26% (95% CI = 0.21 to 0.32, *I*^2^ = 94.7%, *P* < .001). Moreover, the associated factors of anxiety, depression and stress are summarized systematically.

**Conclusion:**

COVID-19 pandemic highly affects mental health of pregnant women in Ethiopia. Anxiety, depression and stress were most reported mental health problems during the pandemic. Appropriate psychological counseling programs should be applied for pregnant women to prevent mental health problems.

## 1. Introduction

Wuhan City, China authorities first reported COVID-19, which was brought on by the severe acute respiratory syndrome coronavirus 2 (SARS CoV-2) in December 2019.^[1]^ Pregnant women have multiple psychological affects and are psychologically fragile.^[2]^ During COVID-19 pandemic, prevalence of depression among pregnant women reached a high level.^[2]^ Female mental health needs to be prioritized above all else if the Sustainable Development Goals are to be met by 2030.^[3]^ Pregnant women had high levels of anxiety and depression symptoms. The 2 main variables linked to psychological distress were found to be concerns about the fetus’s health and fear of infection. Pregnant women were with increased levels of stress, anxiety, and depression during the COVID-19 pandemic.^[4]^ Anxiety caused by corona directly and the fear caused by corona indirectly affecting pregnancy worries are related to the mental health.^[5]^

A study carried out in Shenzhen, China; found that 6.9% and 9.8% of the population, respectively, had symptoms of depression and anxiety. The first trimester of pregnancy, pregnancy complications and vaginal bleeding, unintended pregnancies, decline in household income and arguments between partners brought on by the COVID-19 pandemic, consumption of alcoholic beverages by women and their partners, smoking, lack of exercise, and sedentary lifestyle. Unmarried, divorced, or widowed are factors maximizes the risk of depression and anxiety. Women who have completed junior, high school and college degree had a lower risk of prenatal depression.^[6]^

The scoping review’s conclusions seem to suggest that pregnant women’s levels of stress, anxiety, and depression have increased as a result of the COVID-19 epidemic. The emotional well-being of expectant mothers is greatly influenced by their social support network, educational background, and socioeconomic standing.^[4]^ In fact, because of its undeveloped health systems, weak mental health policies, and inconsistent maternity care, Africa is expected to have catastrophic repercussions from the pandemic on maternal mental health.^[3,7]^ Research conducted in Ethiopia during the COVID-19 pandemic revealed a notable prevalence of mental health issues in expectant mothers. For instance, 45.1% of the expectant mothers reported experiencing psychological anguish. It has a positive correlation with depressive, anxious, and fearful responses to COVID-19.^[8]^

In a similar vein, 34.1% of women said they had prenatal depression. Depression in expectant mothers is associated with the husband’s educational background and divorced status.^[9]^ Anxiety was linked to negative attitudes toward COVID-19 and the belief that the virus posed a significant risk. Anxiety was present in 43.7% of pregnant women.^[10]^ Addressing the mental health needs of pregnant women in emergency situations, pre-hospital emergency systems play a vital role in ensuring the well-being of both mother and baby. It is essential that pregnant women have access to timely and appropriate mental health care during emergencies to promote a healthy pregnancy and childbirth experience.^[11]^

Studies in Ethiopia showed that there was strong evidence of the high prevalence of anxiety, depression and stress among pregnant women during the COVID-19 and findings were heterogeneous. Studying the prevalence and associated factors of mental disorders among pregnant women during COVID-19 in Ethiopia used to understand the impact of the pandemic on maternal mental health and well-being. This information can help to identify at risk populations, inform targeted interventions, and ameliorate overall motherly and child health issues. Also, it can give the long- term implications of the epidemic on maternal mental health and guide future public health policies.

Therefore, a comprehensive study and providing summarized evidence on the prevalence and associated factors of common mental disorders among pregnant women in Ethiopia is not well reported in a summarized way. This study projected to assess the magnitude and associated factors of anxiety, depression and stress among pregnant women during COVID-19 pandemic in Ethiopia.

## 2. Materials and methods

### 2.1. Protocol registration

This study adheres to the PRISMA 2020 guidelines for systematic reviews and meta-analyses.^[12]^ The protocol is registered with PROSPERO under registration number CRD42023389896.

### 2.2. Search strategy

A thorough search for relevant literature was conducted in various databases including PubMed, Cochrane Library, African Journals Online, and Google Scholar. Articles published from December 2019 to August 2024 were included in the review. Observational studies that investigated mental disorders such as depression, anxiety, and stress among pregnant women in Ethiopia during the COVID-19 crisis were targeted. The search process involved using a combination of predefined search terms based on Medical Subject Headings (MeSH) and keywords. Additionally, reference lists of key articles were examined to identify any potentially relevant studies. Duplicates in the search results were eliminated using Mendeley.^[13]^ Two researchers reviewed the titles and abstracts of the studies independently, resolving any disagreements through discussion with a third researcher. The aim was to minimizing the risk of bias in selection and detection. The details of the search strategy can be found in Table 1.

**Table 1:** Search strategy of PubMed database.

Search number	Search detail
#1	“COVID-19” [MeSH Terms]
#2	“mental disorders”[Mesh Terms]
#3	“pregnancy” [Mesh Terms]
#4	“covid 19 pandemic”[Title/Abstract] OR “covid 19 pandemics” [Title/Abstract] OR “covid 19 virus disease”[Title/Abstract] OR “covid 19 virus infection”[Title/Abstract] OR “COVID19”[Title/Abstract] OR “coronavirus disease 2019”[Title/Abstract] OR “coronavirus disease 19”[Title/Abstract] OR “sars coronavirus 2 infection”[Title/Abstract] OR “sars cov 2 infection”[Title/Abstract] OR “severe acute respiratory syndrome coronavirus 2 infection” [Title/Abstract] OR “SARS-CoV-2” [Title/Abstract] OR “2019 novel coronavirus” [Title/Abstract] OR “2019 novel coronavirus”[Title/Abstract] OR “2019- nCoV”[Title/Abstract] OR “covid 19 virus”[Title/Abstract] OR “covid19 virus”[Title/Abstract] OR “Coronavirus disease 2019 virus”[Title/Abstract] OR “SARS coronavirus 2” [Title/Abstract] OR “SARS cov 2 virus”[Title/Abstract] OR “severe acute respiratory syndrome coronavirus 2”[Title/Abstract] OR “Wuhan coronavirus”[Title/Abstract] OR “Wuhan seafood market pneumonia virus”[Title/Abstract] OR “COVID-19”[Title/Abstract] OR “2019 novel coronavirus disease”[Title/Abstract] OR “2019 novel coronavirus infection”[Title/Abstract] OR “2019 ncov disease”[Title/Abstract] OR “2019 ncov infection”[Title/Abstract]
#5	“mental illness” [Title/Abstract] OR “psychiatric problem” [Title/Abstract] AND “anxiety” [Title/Abstract] OR “depression” [Title/Abstract] OR “psychology problem” [Title/Abstract] OR “mental health effect” [Title/Abstract] OR “psychological disturbance” [Title/Abstract] OR “stress” [Title/Abstract] OR “mental disorder” [Title/Abstract] OR “psychiatric Illness” [Title/Abstract] OR “psychiatric diseases” [Title/Abstract] OR “psychiatric disorders” [Title/Abstract] OR “behavior disorders” [Title/Abstract] OR “severe mental disorder” [Title/Abstract]
#6	“pregnant Women”[Title/Abstract] OR “gestation” [Title/Abstract] OR “pregnancies” [Title/Abstract] OR “prenatal”[Title/Abstract] OR “perinatal” [Title/Abstract] OR “postpartum” [Title/Abstract] OR “antenatal” [Title/Abstract] OR “postnatal” [Title/Abstract] OR “puerperal” [Title/Abstract] OR “peurperal” [Title/Abstract] OR “lactating women” OR “reproductive age women” OR “child bearing women” AND “Ethiopia”[Title/Abstract] OR “Addis Ababa”[Title/Abstract] OR “Amhara”[Title/Abstract] OR “Afar”[Title/Abstract] OR “Oromia”[Title/Abstract] OR “SNNP”[Title/Abstract] OR “Somali” [Title/Abstract] OR “Gambella” [Title/Abstract] OR “ Benishangul-Gumuz” [Title/Abstract] OR “Tigrai” [Title/Abstract] OR “ Harari” [Title/Abstract] OR “Dire Dawa” [Title/Abstract]
#7	#1 OR #4
#8	#2 OR #5
#9	#3 OR #6
#10	#7 AND #8 AND #9
#11	Limit to “observational studies” OR “cross-sectional”

SNNP = Southern Nations Nationalities and People of Ethiopia region.

### 2.3. Eligibility criteria

#### 2.3.1. Inclusion criteria

For this study only observational studies/cross-sectional studies examined the prevalence and associated factors of mental disorders among pregnant women during COVID-19 pandemic in Ethiopia were considered. Moreover, this study is follows the condition, context and population (CoCoPop) framework.

***Condition*:** Common mental health problems such as depression, anxiety and stress.

***Context*:** In Ethiopia.

***Population*:** All pregnant women.

***Language***: Studies conveyed in English language were considered.

#### 2.3.2. Exclusion criteria

We excluded the following types of studies: those focusing on the whole population, those lacking sufficient statistical data for extraction, randomized controlled trials, systematic reviews, meta-analyses, editorials, conference abstracts, and opinions.

### 2.4. Outcome measures

The pooled prevalence of depression, anxiety, and stress during COVID-19 among pregnant women in Ethiopia as its primary outcomes. The secondary outcomes included identifying significant factors associated with depression, anxiety, and stress among pregnant women in Ethiopia during the pandemic.

### 2.5. Selection of studies

Two researchers assessed the studies according to specific criteria for eligibility. Initially, they reviewed the titles and abstracts of studies obtained from various databases. Next, a full-text screening process was conducted to examine the complete texts. The rationale for including or excluding studies was presented in the PRISMA diagram.

### 2.6. Data extraction

Data were collected by 2 researchers independently. A pretest of the data extraction form was conducted on 2 pilot studies to ensure all necessary information was collected for the systematic review and meta-analysis. Disagreements were resolved through in-depth discussions. For the included studies, information such as the first author’s last name, publication year, study location, study design, number of cases, sample size, sampling methods, research instruments, study population demographics, average age, prevalence of mental disorders, and associated factors were extracted.

### 2.7. Methodological quality assessment

Two researchers independently assessed the quality of the studies included in the analysis using the Newcastle-Ottawa Scale (NOS).^[14]^ The NOS evaluates studies based on 3 parameters: selection, comparability, and assessment of exposure/outcome. Studies were categorized as low, moderate, or high quality based on their NOS scores of <5, 5 to 7, or more than 7, respectively. Only studies with moderate or high-quality scores were included in the analysis.^[15]^

### 2.8. Data synthesis

The data extracted from Microsoft Excel was imported in to Stata version 14.0 software for the meta-analysis. DerSimonian-Laird random-effects meta-analysis was used to pool the collected data for each outcome with 95% confidence interval (CI). Heterogeneity was assessed using the *I*^2^ statistic and Cochran *Q*-statistic.^[16,17]^ Subgroup analyses were conducted based on regions, year of publication, and sampling methods to identify sources of heterogeneity. Publication bias was evaluated using the Doi plot and Luis Furuya Kanamori (LFK) index, with values outside the −1 to 1 interval indicating asymmetry in the studies.^[18,19]^

## 3. Results

**Figure 1.**
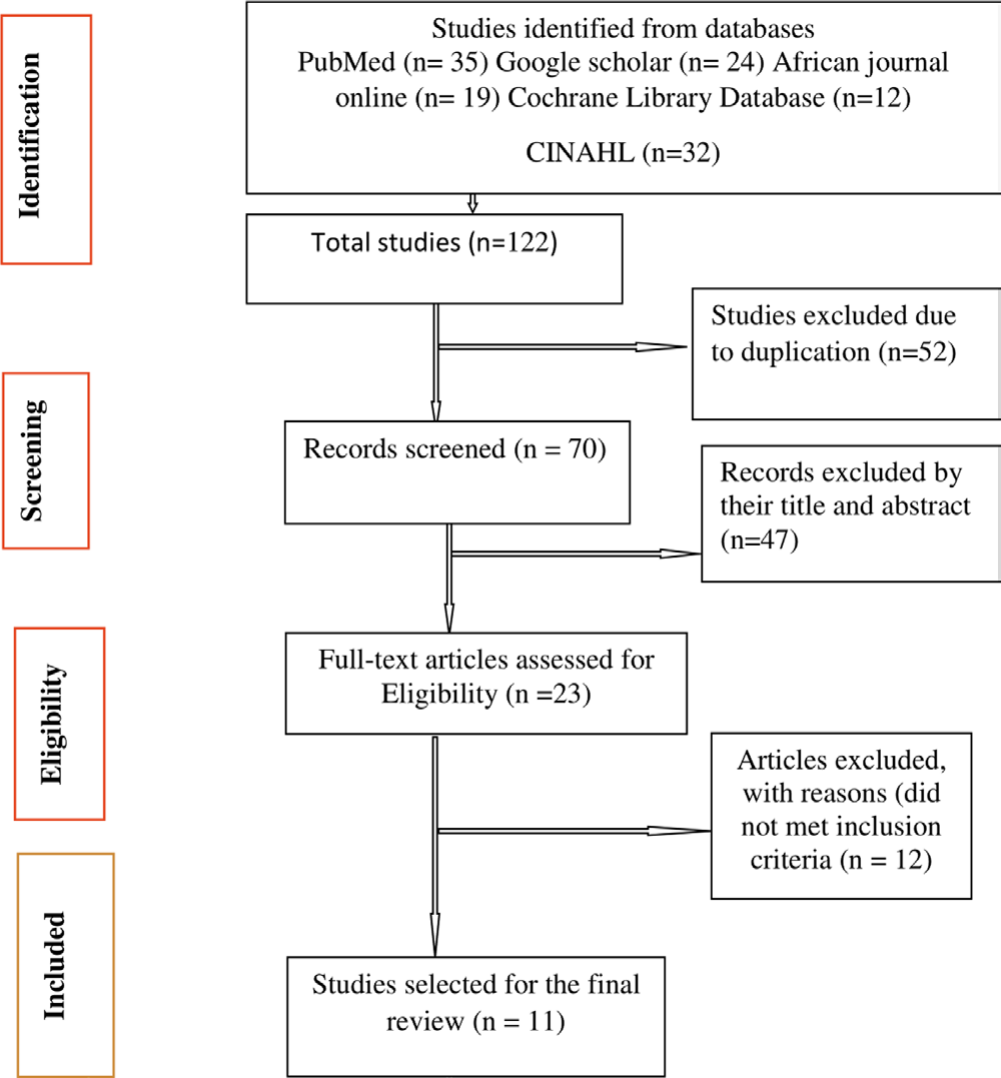
Preferred reporting items for systematic reviews and meta-analyses (PRISMA) flow chart.

A PRISMA diagram showing the steps of database search and refining process for the study on mental disorders of pregnant women during the COVID-19 pandemic was shown in Figure 1. From our databases search, initially 122 studies were identified. 52 duplicates were removed. 47 studies were excluded by checking their title and abstract. 23 full-text studies were examined and 12 studies were removed by reasons that did not met inclusion criteria of COVID-19 era. Finally, we recognized 11 studies appropriate to this systematic review and meta-analysis.

### 3.1. Study characteristics

In this systematic review and meta-analysis, we included 11 cross-sectional studies^[8–10,20–27]^ on the prevalence and associated factors of mental disorders (anxiety, depression, and stress) among pregnant women during COVID-19 pandemic in Ethiopia. Based on the types of mental disorders; 6 studies^[8,10,20,23,24,26]^ reported about anxiety, 5 studies^[8,9,22,23,27]^ reported about depression and 4 studies^[8,21,23,25]^ reported about stress. Furthermore, the summarized data of the key characteristics of the included studies were showed in Table 2.

**Table 2:** Study characteristics and quality of the included studies for depression, anxiety and stress among pregnant women during.

Authors	Year	Region	Mental disorders	Population	Study design	Sampling method	Sample size	Cases	*P* (%)	Instrument	Age (average)	Quality
Dule et al A^[8]^	2021	Oromia	Stress	All PW	CS	Census/consecutive	228	52	22.82	PSS	30.79	7
Dule B^[21]^	2021	Oromia	Stress	All PW	CS	Census/consecutive	384	173	45.1	IES-R	31.30	7
			Anxiety	All PW	CS	Census/consecutive	384	185	48.2	HADS	31.30	
			Depression	All PW	CS	Census/consecutive	384	168	43.8	HADS	31.30	
Molla et al^[22]^	2022	SNNP	Depression	All PW	CS	Multistage	504	87	17.3	EPDS	27.56	8
Amare et al^[9]^	2022	Amhara	Depression	All PW	CS	Systematic	422	144	34.10	DASS-21	28	8
Tibebu et al^[23]^	2022	Amhara	Depression	HIV-positive PW	CS	Systematic	423	159	37.60	DASS-21	29.61	8
			Anxiety	HIV-positive PW	CS	Systematic	423	178	42.1	DASS-21	29.61	
			Stress	HIV-positive PW	CS	Systematic	423	147	34.80	DASS-21	29.61	
Kassaw et al^[24]^	2020	SNNP	Anxiety	PW at perinatal service	CS	Census/consecutive	178	57	32.20	GAD-7	28	7
Biresaw et al^[25]^	2022	Amhara	Stress	All PW	CS	Systematic	415	57	13.70	PSS	28	8
Lelisho et al^[26]^	2022	SNNP	Anxiety	PW at perinatal service	CS	SRS	423	293	69.30	GAD-7	31.09	8
Abate et al^[27]^	2021	Amhara	Depression	HIV-positive PW	CS	Census/consecutive	291	83	28.70	PHQ-9	30.37	8
Abegaz et al^[20]^	2022	Amhara	Anxiety	All PW	CS	Systematic	408	179	43.90	PRAQR	27	8
Bishaw et al^[10]^	2022	Amhara	Anxiety	All PW	CS	Multistage	806	352	43.70	GAD-7	27.57	8

CS = cross-sectional, DASS-21 = 21-item Depression Anxiety Stress Scale, EPDS = Edinburgh Postnatal Depression Scale, GAD-7 = 7-item Generalized Anxiety Disorder scale, HADS = Hospital Anxiety and Depression Scale, IES-R = Impact of Event Scale (Revised), PHQ-9 = 9-item Patient Health Questionnaire, PRAQR = Pregnancy Related Anxiety Questionnaire-Revised, PW = Pregnant women, PSS = Perceived Stress Scale, SRS = simple random sampling, SNNP = Southern Nations Nationalities and People of Ethiopia region, SRS = simple random sampling.

### 3.2. Quality of included studies

A quality score of included studies using the modified Newcastle Ottawa Scale (NOS) for cross-sectional studies quality assessment was presented in Table 2. In this study we considered moderate and high-quality studies for this comprehensive review. Thus, 3 studies were regarded as moderate quality^[8,21,26]^ studies were high quality^[9,10,20,22–25,27]^ were considered for final systematic review and meta-analysis.

### 3.3. Publication bias

**Figure 2.**
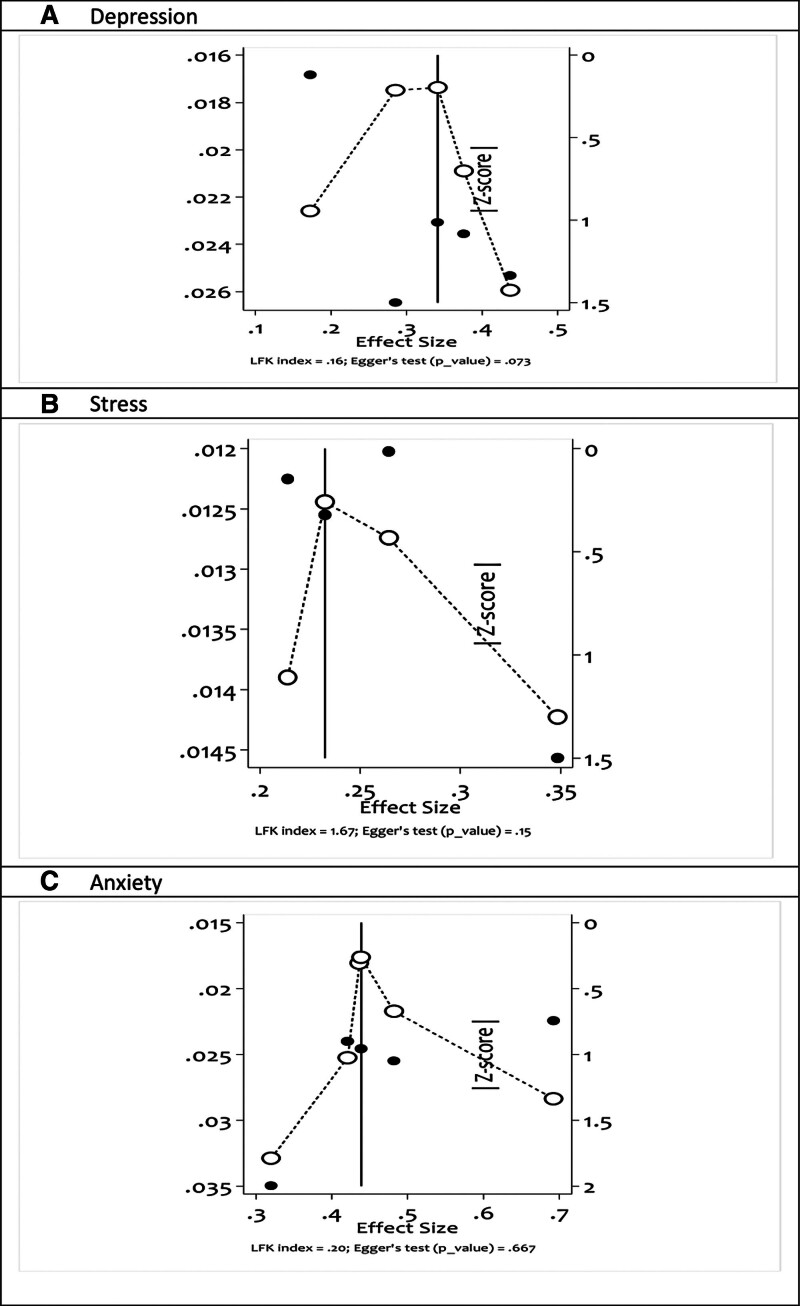
Assessment of publication bias of included studies using Doi plot, LFK index and Egger test.

Publication bias was assessed by the Doi plot,^[18]^ a tool used to visualize asymmetry and by the LFK index,^[19]^ a tool used to detect and quantify asymmetry of study effects. As showed in Figure 2, no publication bias was observed. There is no asymmetry for anxiety studies (LFK index = 0.20, Egger *P*-value = .667), for depression studies (LFK index = 0.16, Egger *P*-value = .073) and for stress studies (LFK index = 1.76, Egger *P*-value = .15). In all cases the LFK index and the statistically insignificant Egger test (*P*-value = .07) supports there is no publication bias for the included studies.^[19]^

### 3.4. Pooled prevalence of anxiety

**Figure 3.**
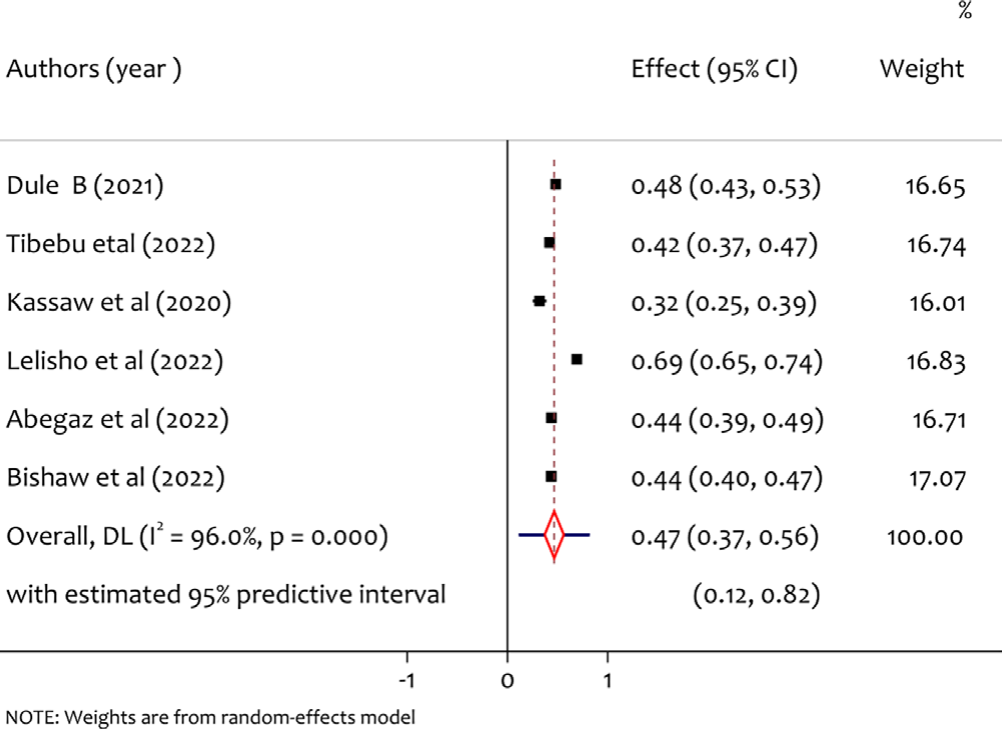
A forest plot for the prevalence of anxiety among pregnant women during the COVID-19 pandemic.

A total of 6 studies reported anxiety and the pooled prevalence of anxiety was found 47% (95% CI: 0.37 to 0.57, *I*^2^ = 96.0%, *P* < .001). As shown in Figure 3, there is significant heterogeneity among study findings. This might be due to a methodological diversity among the included studies.

#### 3.4.1. Subgroup analysis of anxiety by region, year of publication and sampling methods

From the subgroup analysis, prevalence of anxiety by region, the pooled prevalence of anxiety in Oromia, Amhara and Southern Nations Nationalities and People of Ethiopia region (SNNP) region was 48%, 43%, and 51%, respectively (Table 3). The prevalence was higher in SNNP region compared to the others. There was no statistically significant heterogeneity among regions (*Q* = 3.09, *P* = .214).

**Table 3:** Subgroup analysis of mental disorders.

Mental disorders	Subgroup		Pooled prevalence, 95% Confidence Interval	Heterogeneity among subgroups		
				Cochran *Q*-statistics	Df	*P*-value
Anxiety	Region	Oromia	48%, (0.43, 0.53)	3.09	2	*P* = .214
		Amhara	43%, (0.41, 0.46)			
		SNNP	51%, (0.14, 0.87)			
	Year	2020	32%, (0.25, 0.39)	15.06	2	*P* < .001
		2021	48%, (0.43, 0.53)			
		2022	50%, (0.37, 0.62)			
	Sampling design	Census	40%, (0.24, 0.56)	0.22	2	*P* = .894
		Systematic	43%, (0.40, 0.46)			
		Multistage	44%, (0.40, 0.46)			
Depression	Region	Oromia	44%, (0.39, 0.49)	28.38	2	*P* < .001
		Amhara	34%, (0.29, 0.39)			
		SNNP	17%, (0.14, 0.21)			
	Year	2021	36%, (0.21, 0.51)	0.02	1	*P* = .885
		2022	30%, (0.16, 0.43)			
	Sampling design	Census	36%, (0.21, 0.51)	59.65	2	*P* < .001
		Systematic	36%, (0.32, 0.39)			
		Multistage	17%, (0.14, 0.21)			
Stress	Region	Oromia	24%, (0.19, 0.29)	0.615	1	*P* = .419
		Amhara	29%, (0.18, 0.40)			
	Year	2021	24%, (0.19, 0.29)	0.615	1	*P* = .419
		2022	29%, (0.18, 0.40)			
	Sampling design	Census	24%, (0.19, 0.29)	0.615	1	*P* = .419
		Systematic	29%, (0.18, 0.40)			

Df = degree of freedom, SNNP = Southern Nations Nationalities and People of Ethiopia region.

From the subgroup analysis by publication year used (Table 3), the pooled prevalence of anxiety in 2020, 2021, and 2022 was 32%, 48%, and 50%, respectively. The prevalence of anxiety among pregnant women was high in studies published in 2022. The heterogeneity test between group result (*Q* = 15.06, *P* < .001) implies that there was significant heterogeneity in years of publication.

From the subgroup analysis by sampling method used (Table 3), the pooled prevalence of anxiety in studies using census sampling methods was 40%, systematic sampling was 43% and multistage sampling 44%. The heterogeneity test between group result (*Q* = 0.22, *P* = .894) implies that there is no statistically significant heterogeneity among sampling methods used.

### 3.5. Pooled prevalence of depression

**Figure 4.**
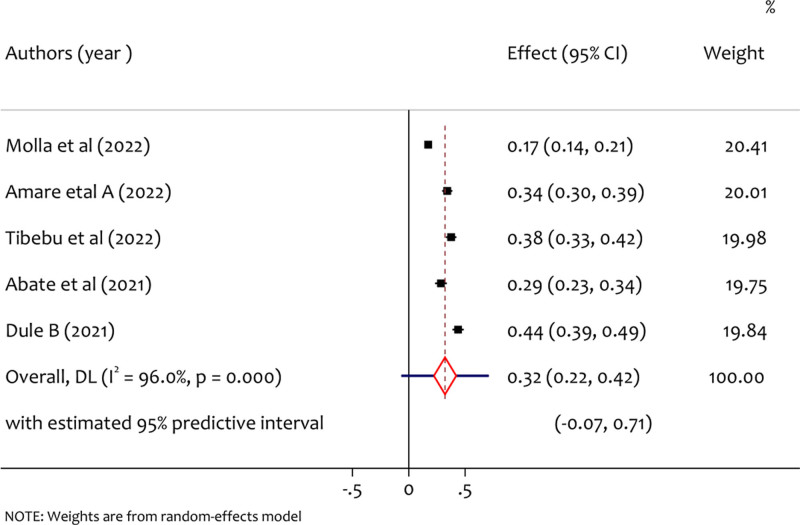
A forest plot for the prevalence of depression among pregnant women during the COVID-19 pandemic.

From a total of 5 studies, the pooled prevalence of depression was 32% (95% CI = 0.22 to 0.42, *I*^2^ = 96.0%, *P* < .001) as shown in the forest plot Figure 4. There is significant heterogeneity on study results has been observed since *I*^2^ = 96.0%. This might be due to there is a methodological diversity among the included studies.

#### 3.5.1. Subgroup analysis of depression by region, year of publication and sampling methods

From the subgroup analysis of prevalence of depression by region, the pooled prevalence of depression in SNNP was 17%, in Amhara was 34% and in Oromia was 44% (Table 3). The prevalence was higher in Oromia region compared to the others. There was statistically significant heterogeneity among regions (*Q* = 83.76, *P* < .001). This variance might be due to the differences in level of awareness among pregnant women regarding COVID-19 pandemic across regions.

From the subgroup analysis by publication year (Table 3) the pooled prevalence of depression in 2021 was 36% and 30% in 2022. The prevalence of depression among pregnant women was high in studies published in 2021. The heterogeneity test between group result (*Q* = 0.41, *P* = .520) implies that there was no statistically significant heterogeneity between years of publication on the prevalence of depression.

From the subgroup analysis by sampling methods (Table 3) the pooled prevalence of depression in study using sampling methods multistage sampling, systematic sampling, and census was 17%, 36%, and 36%, respectively. The heterogeneity test between group result (*Q* = 60.69, *P* < .001) implies that there is statistically significant heterogeneity among sampling methods used. This heterogeneity might be due to the difference in the nature of sampling methods on the selection of samples to make inferences.

### 3.6. Pooled prevalence of stress

**Figure 5.**
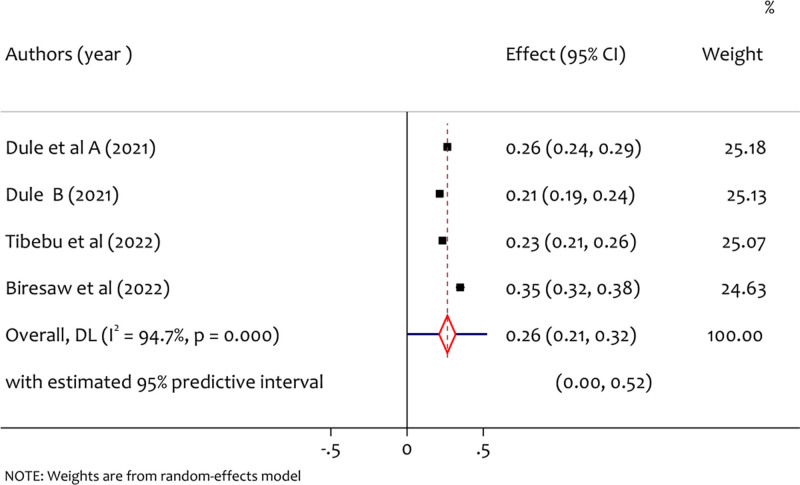
A forest plot for the prevalence of stress among pregnant women during the COVID-19 pandemic.

From a total of 4 studies, the pooled prevalence of stress was 26% (95% CI = 0.21 to 0.32, *I*^2^ = 94.7%, *P* < .001) as shown in the forest plot Figure 5. The significant variability of study results has been observed since *I*^2^ = 94.7%. This might be due to there is a methodological diversity among the included studies.

#### 3.6.1. Subgroup analysis of stress by region, year of publication and sampling methods

From the subgroup analysis of prevalence of stress by region, the pooled prevalence of stress in Oromia and Amhara was 24% and 29%, respectively (Table 3). The prevalence was higher in Amhara region compared to Oromia. There was no statistically significant heterogeneity between regions (*Q* = 0.615, *P* = .419).

From the subgroup analysis by publication year of publication (Table 3) the pooled prevalence of stress in 2021 and 2022 was 24% and 29%, respectively. The prevalence of stress among pregnant women was high in studies published in 2022. The heterogeneity test between group result (*Q* = 0.615, *P* = .419) implies that there was no significant heterogeneity in years of publication on the prevalence of stress among pregnant women.

From the subgroup analysis by sampling methods (Table 3), the pooled prevalence of depression in study using census sampling methods and systematic sampling was 24% and 29%, respectively. The heterogeneity test between group result (*Q* = 0.59.65, *P* = .419) implies that there is no statistically significant heterogeneity among sampling methods used.

### 3.7. Associated factors of mental disorders among pregnant women during COVID-19

As shown in Table 4, we have summarized systematically the associated factors of mental disorders among pregnant women during the COVID-19 pandemic in Ethiopia.

**Table 4:** A summary reviews of significant associated factors of mental disorders among pregnant women during the COVID-19.

Authors (year)	Mental disorders	Associated factors	Adjusted odds ratio, 95% CI
Amare et al (2022)^[9]^	Depression	Divorced marital status	7.52 (2.70, 20.91)
		Can read and write without formal education	2.39 (1.10, 5.15)
Tibebu et al (2022)^[23]^	Depression	Having an HIV-negative sexual partner	1.91 (1.16, 3.15)
		Being on antiretroviral therapy >1 year	2.18 (1.41, 3.36)
Tibebu et al (2022)^[23]^	Anxiety	Unplanned pregnancy	1.09 (1.02, 2.33)
		Did not discuss with the sexual partner about HIV	3.21 (2.12, 7.07)
Kassaw et al (2020)^[24]^	Anxiety	Living in rural area	0.48 (0.25, 0.90)
		Primary level of education	0.41 (0.21, 0.75)
		Poor social support	4.39 (2.29, 12.53)
		Primigravida	3.05 (1.53, 6.08)
Biresaw et al (2022)^[25]^	Stress	Being a student	9.67 (2.73, 34.18)
		Being at the first trimesters	3.56 (1.06, 11.88)
		Third trimesters	4.80 (1.85, 14.47)
		Having antenatal depression	3.51 (1.62, 7.56)
Lelisho et al (2022)^[26]^	Anxiety	Urban residents	1.82 (1.23, 2.70)
		Having alcohol habit	3.437 (1.39, 8.45)
		Having occupation	0.50 (0.30, 0.85)
		Being health care worker	0.11 (0.04, 0.31)
		Having chronic illness	7.68 (3.04, 19.39)
		Having family history of anxiety/mood disorder	7.83 (2.656, 23.12)
		Fear of contracting COVID-19	1.70 (1.15, 2.52)
		Having moderate social support	0.64 (0.42, 0.98)
Abate et al (2021)^[27]^	Depression	Age ≥30 years	1.32 (1.24, 3.35)
		Urban residency	1.76 (1.57, 4.61)
		Having first pregnancy <18 years	3.82 (1.54, 17.34)
		Known HIV sero-status during pregnancy	1.29 (1.08, 2.47)
		Have COVID-19-related knowledge	0.32 (0.12, 1.12)
Abegaz et al (2022)^[20]^	Anxiety	Having no formal education	3.37 (1.32, 8.58)
		Primigravida	1.94 (1.17, 3.24)
		Intimate partner violence	2.88 (1.47, 5.64)
		Poor social support	2.05 (1.18, 3.56)
Bishaw et al (2022)^[10]^	Anxiety	Having <3 the number of children	1.53 (1.11, 2.13)
		Having a negative attitude about COVID-19	1.47 (1.07, 2.02)
		Having a high-risk perception about COVID-19	1.86 (1.34, 2.57)

## 4. Discussion

This comprehensive study aimed to assess the impact of COVID-19 pandemic on anxiety, depression and stress among pregnant women in Ethiopia. Studies showed that a number of pregnant women developed variety of mental health problems due to COVID-19 pandemic. There are studies at the single level, but to our knowledge, this systematic review and meta-analysis is the first of its kind that assessed the pooled prevalence and associated factors of anxiety, depression and stress among pregnant women during COVID-19 in Ethiopia. In this study, the pooled prevalence and associated factors of anxiety, depression and stress were assessed. This study includes 11 articles focusing on the impact of COVID-19 on common mental disorders among pregnant women in Ethiopia. The pooled magnitude of common mental disorders such as anxiety, depression and stress, and their associated factors were discussed. In Ethiopia, during the COVID-19 pandemic, 47% of pregnant women experienced anxiety. This is higher than the global rate of 32.8%^[27]^ and higher than rates found in studies from India which ranged from 35.8% to 37%.^[28,29]^ However, it is lower than the prevalence of anxiety found in studies from Egypt, which was 73%,^[30]^ and study result worldwide pooled prevalence was 42%^[31]^ and 43.3%.^[32]^ From the subgroup analysis by region, the pooled prevalence of anxiety in Oromia, Amhara and SNNP was 48%, 43%, and 51%, respectively. The prevalence was higher in SNNP region compared to the others. This variation might be due to the difference on the level of awareness about COVID-19 pandemic among pregnant women in regions. From subgroup analysis by year of publication, the pooled prevalence of anxiety in 2020, 2021, and 2022 was 32%, 48%, and 50%, respectively. This shows the prevalence was high in 2021 and 2022.

The pooled prevalence of depression among pregnant women during the COVID-19 pandemic was 32%, this study is in line with the meta analysis result prevalence was 31%,^[28]^ higher than meta-analysis results the prevalence depression was (24.9%),^[29]^ 25%,^[30]^ 6.7%,^[31]^ worldwide meta-analysis prevalence report 27.8%,^[32]^ and lower than in Egypt prevalence was 68.5%.^[33]^ The subgroup analysis by region, the pooled prevalence of depression in SNNP was 17%, in Amhara was 34% and in Oromia was 44%. The prevalence was higher in Oromia region compared to the others. The variation among regions on the prevalence might be due to the difference on the level of awareness about COVID-19 pandemic among pregnant women across regions. From the subgroup analysis by publication year the pooled prevalence of depression in 2021 was 36% and 30% in 2022. There was no statistically significant heterogeneity between years of publication on the prevalence of depression.

The pooled prevalence of stress among pregnant women during the COVID-19 pandemic was 26%. This was almost in line with the meta analysis prevalence of stress was 29.4%,^[29]^ lower than study in Egypt prevalence of stress was 61.5%.,^[33]^ and meta-analysis report the prevalence was 70%,^[28]^ 56%.^[34]^ From the subgroup analysis of prevalence of stress by region the pooled prevalence of stress in Oromia and Amhara was 24% and 29%, respectively.

The discussion surrounding the factors contributing to the likelihood of developing anxiety in urban residents, those with an alcohol habit, chronic illness, and family history of anxiety/mood disorders are important considerations in understanding the prevalence of anxiety in pregnant women during COVID-19 in Ethiopia. Research has shown that individuals living in urban areas may face added stressors such as noise pollution, overcrowding, and lack of access to green spaces, which can increase the risk of developing anxiety. Additionally, individuals with chronic illnesses may experience heightened levels of anxiety due to the challenges of managing their condition on a daily basis. Individuals with a family history of anxiety or mood disorders are more likely to inherit a genetic predisposition to developing anxiety themselves. This highlights the importance of understanding one’s family history and taking proactive steps to address mental health concerns. On the other hand, the study^[10]^ reported that variables such as having <3 children in the household, a negative attitude about COVID-19, and a high-risk perception about COVID-19 can also contribute to the likelihood of developing anxiety. The relationship between social support and occupation with the likelihood of developing anxiety is an important factor to consider. Research has shown that having moderate social support and being employed can decrease the likelihood of developing anxiety. This highlights the importance of having a strong support system and a sense of purpose or fulfillment in one’s daily life. On the other hand, factors such as being a first-time pregnant woman, experiencing intimate partner violence, and lacking in social support can increase the likelihood of developing anxiety.^[20]^ Depression of pregnant women also affected by several factors. The study on HIV-positive pregnant women highlights factors that increase the likelihood of developing depression. Being divorced, having a husband who cannot read and write, having an HIV-negative sexual partner, and being on antiretroviral therapy for more than 1 year were all associated with a higher risk of depressive symptoms.^[23]^ On the other hand, factors such as age over 30 years, living in an urban area, and having knowledge about COVID-19 were found to decrease the likelihood of developing depression.^[27]^ These findings suggest that social and health-related factors play a crucial role in the mental health outcomes of pregnant women. It is important for healthcare providers to take these factors into consideration when assessing and managing the mental health needs of this vulnerable population during the pandemic situations.

The impact of stress on pregnant women during the COVID-19 pandemic is a crucial topic that requires attention and consideration. The study conducted in Ethiopia sheds light on the associated factors of stress among pregnant women, highlighting the importance of addressing these factors to ensure the well-being of both the mother and the unborn child.^[25]^ One significant finding of the study is the correlation between different stages of pregnancy and the likelihood of developing stress. Pregnant women in their first and third trimesters are at a higher risk of experiencing stress, which can have adverse effects on their mental and physical health. It is essential for healthcare providers and support systems to be aware of this increased vulnerability during these stages of pregnancy and provide appropriate interventions and support to mitigate the stress. Another factor identified in the study is the presence of antenatal depression, which can further exacerbate stress levels among pregnant women. Antenatal depression is a serious condition that requires prompt attention and treatment to prevent negative outcomes for both the mother and the baby. Health professionals should be vigilant in screening for antenatal depression and providing necessary interventions to support pregnant women in coping with their mental health challenges. Additionally, the study highlights the role of being a student as a factor that contributes to stress among pregnant women during the pandemic. The unique challenges faced by pregnant student mothers, such as balancing academic responsibilities with pregnancy and the added stress of navigating the uncertainties brought about by COVID-19, can significantly impact their mental well-being. Moreover studies supports that the burden of caring for a family member with COVID-19 may compound these mental health challenges for pregnant women. It is important for healthcare providers to be aware of these unique challenges faced by pregnant women caring for family members with COVID-19, and to provide appropriate support and resources to address their mental health needs of pregnant women^[35]^ In reducing symptoms of stress, anxiety, and depression in pregnant women in critical situations it is recommended using strategies to promote socioeconomic status can play an effective role in controlling common mental health problems in pregnant women.^[36]^

Although, this study is with strengths and limitation. This review is the first of its kind that reported the pooled prevalence and associated factors of anxiety, depression and stress among pregnant women during COVID-19 in Ethiopia. This population group is with a disproportionate burden of psychological problems even before COVID-19. Searching, screening, data extraction and methodological quality assessment were done by 2 researchers independently. The NOS was used to assess the quality of included studies were strengths. The absence of sufficient studies in all regions of Ethiopia on the mental health impact of COVID-19 among pregnant women which could impact on the overall estimated prevalence rate, there is a methodological diversity among the included studies, there is a significant heterogeneity in this meta-analysis, which might affect results and their generalization, all studies included in this meta-analysis were cross-sectional, which only account for prevailing circumstances, thereby lacking a longitudinal aspects. Studies included were only published in the English language, which might have a language bias are limitations of the study.

## 5. Conclusion

Pregnant women in Ethiopia were affected by a variety of mental disorder during COVID-19 pandemic. The prevalence of anxiety, depression and stress among pregnant women were significantly high in Ethiopia during COVID-19 pandemic. The timely establishing programs on psychological counseling and intervention should have been applied for pregnant women to improve mental health during this pandemic and future public health crisis. Future studies should investigate the impact of preexisting mental health conditions on the prevalence of common mental disorders during pregnancy in the context of COVID-19 in Ethiopia. Explore the role of social support systems and access to mental health services in mitigating the risk of common mental disorders among pregnant women during the COVID-19 pandemic. Moreover studies on mental disorders among pregnant women should be well reported by considering a longitudinal aspect to meet temporality during the pandemic situations.

## Acknowledgments

The author’s acknowledged researchers of the primary studies included for this review.

## Author contributions

**Conceptualization:** Aragaw Asfaw Hasen.

**Data curation:** Aragaw Asfaw Hasen, Ahmed Adem Mohammed, Mekash Ayalew Mohammed, Abubeker Alebachew Seid.

**Formal analysis:** Aragaw Asfaw Hasen, Ahmed Adem Mohammed, Mekash Ayalew Mohammed, Abubeker Alebachew Seid.

**Funding acquisition:** Aragaw Asfaw Hasen, Ahmed Adem Mohammed.

**Investigation:** Aragaw Asfaw Hasen, Mekash Ayalew Mohammed, Abubeker Alebachew Seid.

**Methodology:** Aragaw Asfaw Hasen, Mekash Ayalew Mohammed, Abubeker Alebachew Seid.

**Project administration:** Aragaw Asfaw Hasen.

**Resources:** Aragaw Asfaw Hasen, Ahmed Adem Mohammed, Mekash Ayalew Mohammed, Abubeker Alebachew Seid.

**Software:** Aragaw Asfaw Hasen.

**Supervision:** Aragaw Asfaw Hasen.

**Validation:** Aragaw Asfaw Hasen, Ahmed Adem Mohammed, Mekash Ayalew Mohammed.

**Visualization:** Aragaw Asfaw Hasen, Ahmed Adem Mohammed, Mekash Ayalew Mohammed, Abubeker Alebachew Seid.

**Writing – original draft:** Aragaw Asfaw Hasen, Ahmed Adem Mohammed, Mekash Ayalew Mohammed, Abubeker Alebachew Seid.

**Writing – review & editing:** Aragaw Asfaw Hasen, Ahmed Adem Mohammed, Mekash Ayalew Mohammed, Abubeker Alebachew Seid.
